# Supplementary material for: Toxoplasma gondii infection and insomnia: A case control seroprevalence study
Source: PLoS One. 2022 Jun 9;17(6):e0266214. doi: 10.1371/journal.pone.0266214 (PMC9182222; doi:10.1371/journal.pone.0266214)
Supplement: Attachment — Submitted filename: Response to reviewers Tg-Insomnia.docx [file pone.0266214.s002.docx]

**RESPONSE TO THE REVIEWERS’ COMMENTS**

Journal Requirements:

1. Please ensure that your manuscript meets PLOS ONE's style requirements, including those for file naming.

**The manuscript was modified according to the PLOS ONE’s style requirements.**

2. If you have no competing interests, please state "The authors have declared that no competing interests exist.", as detailed online in our guide for authors at http://journals.plos.org/plosone/s/submit-now

**The cover letter includes the following statement: “The authors have declared that no competing interests exist”.**

**Thank you for your valuable comments for improving our manuscript.**

Reviewer #1:

1. The approval ethical number needs to add.

**The approval ethical number was added (line 129).**

2. The authors only checked the serum samples with anti-T. gondii IgG antibodies for analyze and detection of anti-T. gondii IgM antibodies so may be lost the samples with IgM positive and IgG negative.

**The presence of anti-*T. gondii* IgM antibody was determined only in anti-*T. gondii* IgG positive samples. The presence of IgM without IgG is not a reliable result because enzyme immune assays for anti-*T. gondii* IgM antibodies have high numbers of false-positive results. This information was added to the Discussion section (lines 221-224).**

3. The data of table 1 is the positive of T. gondii is related to IgG or IgM results.

**It is related to IgG antibodies. This information was added to the Title of Table 1.**

4. There is not any tables about IgM results.

**Since there was no difference in the frequency of IgM antibodies among groups, no further analysis of IgM results in a Table was performed.**

5. Is T. gondii related to the kind of symptoms: 1-difficulty initiating sleep, 2- difficulty maintaining sleep and 3- early-morning awakening with inability to return to sleep, or no?

**We did not obtain this information. Studies to determine an association between *T. gondii* infection and specific symptoms of insomnia are needed. This information was added to the Discussion section (lines 224-226).**

6. The duration of this study from 2014-2019 is very wide period and this may be affected the age grouping.

**We further evaluate the association between *T. gondii* seropositivity and insomnia by adjustment for age. This information was added to the Materials and methods section (lines 121-123), Results section (lines 155-157), and Discussion section (lines 183-185).**

7. All sera that collected during 5 years were evaluated for IgG and IgM against T. gondii in the same time or no.

**Sera were analyzed every few months during the study period. This information was added to the Materials and methods section (line 108).**

**Thank you for your valuable comments for improving our manuscript.**

Reviewer #2:

1. Introduction

l.88 : « responsible » there is no link of causality that has been clearly demonstrated, only statistical association

**The word “responsible” was deleted. The sentence was rewritten as follows: ”Chronic toxoplasmosis might be associated with a…” (line 62).**

2. l90-95: As mentioned by the authors several studies have already looked into this association with conflicting results, however article by Corona C should be cited and discussed (Toxoplasma gondii IgG associations with sleep-wake problems, sleep duration and timing, Pteridines. 2019 Feb; 30(1): 1–9.). Furthermore we believe that the only fundamental study in a mice model should also be included and discussed (Dupont et al, Chronic Toxoplasma gondii infection and sleep-wake alterations in mice, CNS Neurosci Ther. 2021 Aug;27(8):895-907).

**Our results were contrasted with those reported by Corona et al, and Dupont et al, (lines 203-206, and lines 218-220, respectively).**

3. Methods

The heterogeneity of symptoms associated with insomnia should also be commented on in the discussion.

**We did not obtain this information. Studies to determine an association between *T. gondii* infection and specific symptoms of insomnia are needed. This information was added to the Discussion section (lines 224-226).**

4. Selection of controls: it is known that T. gondii seroprevalence is strongly influenced by education or residence? Thus are controls similar to cases on these parameters? If no information available, this should be discussed.

**Information about education and residence in participants was added to Table 1. We further evaluate the association between *T. gondii* seropositivity and insomnia by adjustment for age, education and residence. This information was added to the Materials and methods section (lines 121-123), Results section (lines 155-157), and Discussion section (lines 183-185).**

5. Furthermore it is known that sleep is an index of brain functions as sleep alteration are usually observed in several neuropsychiatric or neurodegenerative disorders even before clinical neurological manifestations. If controls/cases were selected when attending medical institution, did these patients were screened for these disorders associated with sleep alterations? this should also be discussed.

**This was a limitation of the study. We did not screen for neuropsychiatric or neurodegenerative disorders among participants. Further studies with information about sleep clinical variables and the presence of neuropsychiatric or neurodegenerative disorders among participants are needed (lines 226-229).**

6. Age groups are not described in the methods, we believe that analyzing the effects of age as a continuous variable should be better thant categorizing by arbitrary age groups.

**We further evaluated the association between *T. gondii* seropositivity and insomnia by adjustment for age. This information was added to the Materials and methods section (lines 121-123), Results section (lines 155-157), and Discussion section (lines 183-185).**

7. Laboratory tests

Were the analyses performed blindly as regards to the staus of cases/controls ?

**Laboratory tests were performed blindly, the analyst did not have information about the history of insomnia in participants during the analysis of samples. This information was added to the Materials and methods section (lines 110-112).**

8. Was there a grey zone in the assay used ? if so how was it further processed ?

**Samples with results just below 8 IU/ml (grey zone) or clearly lower were considered as negatives (lines 104-105).**

9. Discussion

Reference 18 does not conclude to an association however, there is statistical trend toward shorter sleep duration in seropositive men. This should be added to the discussion, in line with results from Dupont et al, Chronic Toxoplasma gondii infection and sleep-wake alterations in mice, CNS Neurosci Ther. 2021 Aug;27(8):895-907.

**Information about the secondary analysis (reference 18) that showed a trend toward shorter sleep duration in seropositive men was added in the Discussion section (lines 201-203).**

10. Furthermore, it should be clearly stated that this study does not allow to conclude that T. gondii leads to insomnia. Statistics do only point towards an association without precluding the direction of the association, all the most if patients were not selected as free of neuropsychiatric or degenerative disorders. Cinetics is of utmost importance and cinetics cannot be performed when using only one serum.

**We added the following information: Results of our study do not mean that *T. gondii* infection leads to insomnia. Only longitudinal studies may help to determine the direction of the association (lines 229-231).**

**Thank you for your valuable comments for improving our manuscript.**

Reviewer #3:

1. I suggest that the authors could enhance the usefulness of this work for the community if they redesign the tables.

**Table 1 was modified. Information about sociodemographic variables was added.**

**Thank you for your valuable comments for improving our manuscript.**
